# The German pelvic database

**DOI:** 10.1007/s00402-025-05782-3

**Published:** 2025-02-19

**Authors:** Tim Pohlemann, Axel Gänsslen

**Affiliations:** 1 Universitätsklinikum des Saarlandes, Homburg, Germany; 2 Hannover Medical School, Hanover, Germany

**Keywords:** Data base, Multicenter study, German pelvic group, Lessons

## Abstract

The German Pelvic Group as part of the former German section of the AO-International (now AO Trauma Germany) and the German Trauma Society (DGU) represents a 34-year success story of data documentation for the optimization of pelvic and acetabulum surgery. The historical development and the corresponding course are presented. With increasing work, the initial data were integrated into the DGU Pelvic Register. It is used to record data on fractures of the pelvic ring and acetabulum with the aim to derive optimized treatment options and to gain new scientific knowledge. The register started in 2004 as an initiative of the DGU GPG. In June 2024, the DGU Board approved the upgrade of the status of the working group to a formal section Pelvic and Acetabulum Fractures as standing division of the German Trauma Society.

## Introduction

In 1989, Harald Tscherne, Trauma Director at the Hannover Medical School (MHH) noticed some discrepancies in the statistics of the trauma departments patients after pelvic ring injuries and acetabular fractures reaching back to the inauguration of this first academic Trauma unit in 1970. He delegated the task to get a solid overview about the results to Tim Pohlemann, just having finished his residency in General Surgery. With a team of students and enthusiasts under the lead of undergrade medical school student Axel Gänsslen, they reviewed all accessable files and radiographs to end up with a series of 1500 P/A cases, later published in CORR [8].

**Fig. 1:**
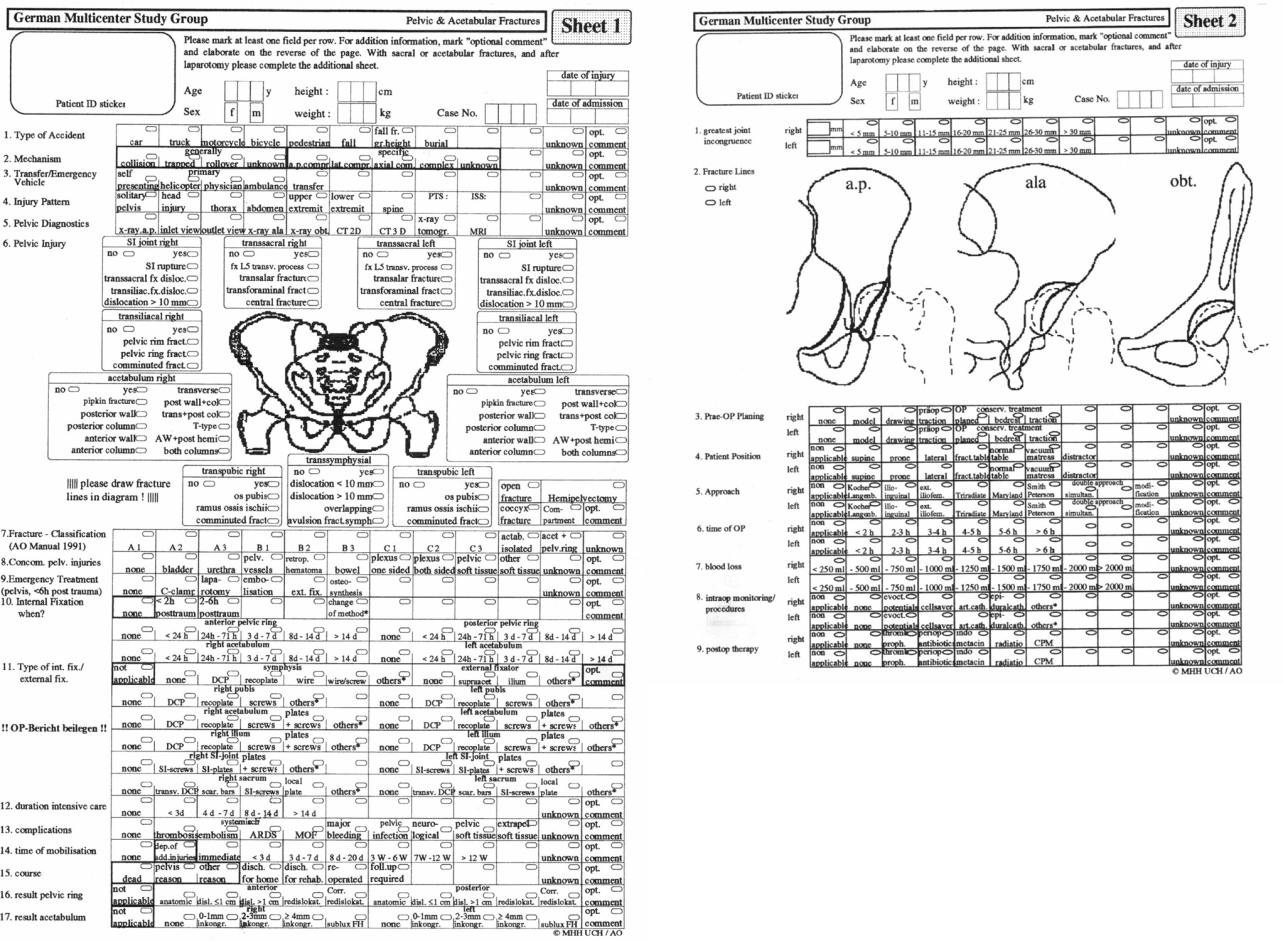
The initial data collection sheets 1990 with integration of a schematic pelvic x-ray: pelvic ring and acetabular documentation

**Fig. 2:**
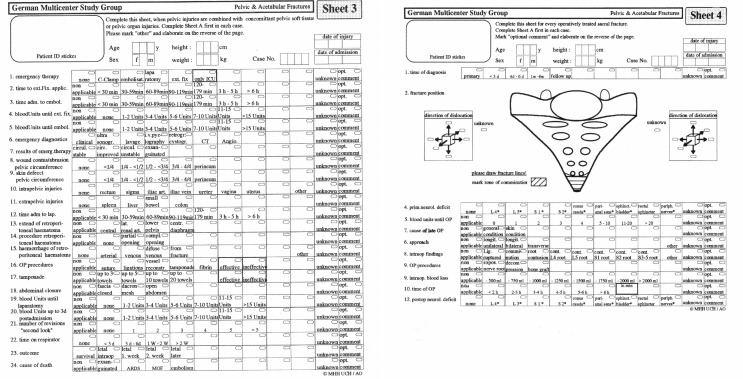
The initial data collection sheets with integration of a schematic pelvic x-ray: emergency treatment and specific sacral fracture documentation

Forward looking a data base was programmed (Filemaker^®^, Filemaker Pro^Ⓡ^) which allowed prospective documentation of a wide range of basic and advanced parameters. Of interest, already with the very limited storage space not allowing to integrate the still analog radiographs, a small drawing of an a.p. pelvis and oblique hip images allowed the demonstration of the individual fracture lines (Figs. 1 and 2). In the use of the data base this turned out to be one of the most important factors of quality assurance and data consistency.

In 1992, with the use of research grants the “Hannover Pelvic Group” invested (> 20.000 DM) in one of the early digital cameras for replacing the still analog image collection on slides and x-ray films. The Kodak DCS 200 series, introduced in 1992, condenses the storage unit into a module which is mounted onto the base and back of a stock Nikon 8008 SLR film camera. It was the first digital camera to use the Bayer color filter pattern. The module contains a built-in 80 MB hard drive and was powered with AA batteries [2].

Being not only trauma, but as well, with increasing passion, digital enthusiasts, the database was enhanced by modular expansion of image data-bases, linked to the case and automated options for image processing, optimization and measurement [10].

But the reality of the digital world in those days was fairly sobering, especially for the students in charge of the forward and backward documentation! Every weekend, one of the young group members started a search mission on Friday afternoon exploring the central x-ray archive (good connection to the radiology technicians mandatory!) to collect uncountable x-ray envelopes from the radiological department, bring them in the highly secured digitalization room, sorting hundreds of images of the frequent polytraumatized patients and doing manual digitalization of every single radiograph, frequently with focus and detailed magnification. This “picture-taking” fulfilled the whole weekend until late Sunday evening, when all x-ray enveloped had to be brought back to the archive and had to find their way back in the right space in the racks. With this tremendous effort lasting 3 years all retrospective and the actual prospective cases series were digitized completely, resulting in complete files for 1578 patients with pelvic and acetabular fractures until 1992.

This experience was shared and transferred to the German Multicenter Study Group, being initiated in November 1989 under the umbrella of the German Section of the AO International and the German Trauma Society DGU. Therefore, the Hannover series can be considered the basis and foundation of the present German P/A registry, now connected to the national DGU Trauma Registry [8, 11].

## Historical evolution of the German Pelvic Group (GPG)

In 1990, during the summer meeting of the German AO the unsatisfactory study situation and poor treatment quality regarding pelvic and acetabular fractures was addressed. The AO in Germany already had conducted a multicenter data collection on this topic under the lead of Karl-Heinz Jungbluth, but the results were outdated and not reflecting the actual possibilities in the growing field of P/A reconstruction. Harald Tscherne was asked by the Board to initiate another, specific, multicentre working group and he commissioned Tim Pohlemann by telephone to quickly develop proposals for the structure of a pelvic working group and reasonable parameter for a study protocol concentrating on the present reality of care and treatment outcomes after pelvic and acetabular fractures in Germany. The fact that the order was placed by telephone, an extremely unusual circumstance, indicated to Tim Pohlemann considerable urgency. He was just in the middle of a research fellowship concentrating on treatment options for sacrum stabilization together with Reinhold Ganz.

**Fig. 3:**
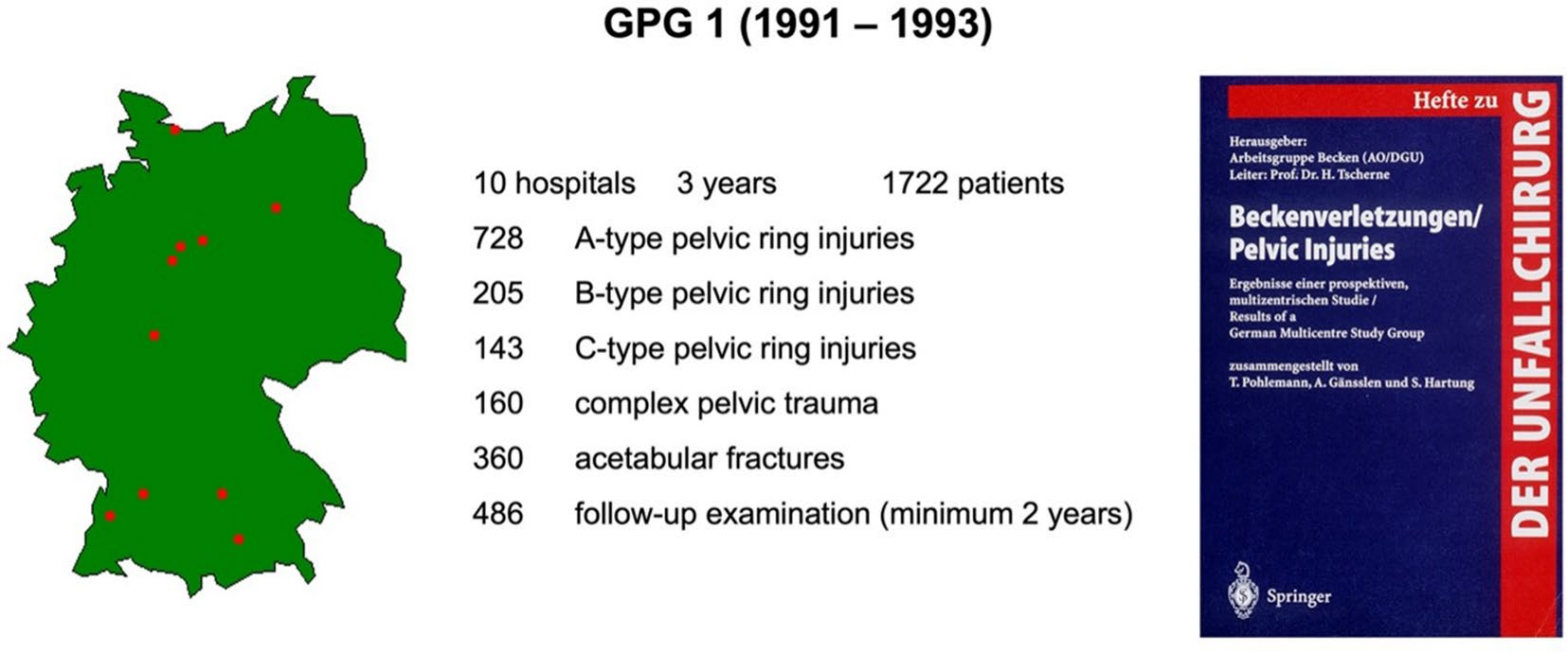
GPG 1 with analysis of 1722 patients resulting in a detailed book on pelvic and acetabular fracture data

In January 1991, the data collection began at ten large German trauma hospitals (Fig. 3). On request of Professor Tscherne and Tim Pohlemann, the AO working group was additionally accredited by the German Society of Trauma Surgery (DGU) at the DGU Board meeting in summer 1991 and was now fully operational.

The result was a success story that was unique at that time and which contributed significantly to the improved cooperation of the German Trauma Departments. The establishment of a pelvic registry has provided a relevant impulse to the reality and quality of treatment after pelvic and acetabular fractures up to today. Parallel, data collections for polytraumatized patients, spine and pelvic injury patients were initiated which led to the worldwide unique structure of quality control in trauma care in Germany (German Trauma Network, German Trauma Registry and others).

The German Pelvic Group was also able to overcome long-standing rivalry between the leading trauma hospitals in Germany at that time and traditionally since the beginning in the early 90s inspired young trauma surgeons for the topic and the systematic clinical research on treatment results in P/A to improve the standard of care. The close cooperation of the members in difficult cases and the mutual trust promotes the exemplary, widespread cooperation. In the early days, complex pelvic trauma dominated the discussion, which at the time had a mortality rate of over 50% which could be reduced to 15% [8, 12]. Demographic change has now brought geriatric injuries into a dominant focus.

The initiated GPG worked scientifically, published regularly, is internationally recognized and is involved in the scientific program of the annual trauma congress. It is dedicated to a wide variety of other topics.

### The 1st German Pelvic Group (GPG 1; chairman: Prof. T. Pohlemann)

The initiation of the GPG 1 was based on the Hannover Medical School pelvic database experience.

The use of a self-programmable database (Filemaker^®^) was very modern and ran on an Apple Macintosh computer with an integrated possibility of machine-readable data capture, which was already implemented from the AO for their fracture classification database. By encoded integration of image information in forms of drawings of the fracture lines, classification errors could be effectively identified and corrected after consultation.

For instance, some both column fractures were coded as complete pelvic ring ilium fractures with an associated anterior ring fracture and an acetabulum fracture leading to a classification of a type C pelvic ring injury and a combined acetabular fracture, but was exclusively an associated both column fracture.

The GPG 1 data set, which was developed on the basis of the Hannover data, was based on the AO data collection forms and focused on general information/demography, pelvic ring injuries, acetabular fractures, complex pelvic trauma, sacral fractures and pediatric fractures.

The enthusiasm and activity in this group was infectious. Even students, young doctors and heads of trauma departments had intensive discussions during the annual two-day working meetings. In addition to the scientific and administrative consultations, practical elements for training, development and exchange of experience were always offered, which ultimately led to an extremely collegial and trusting interaction across many generations of surgeons that has been maintained until today.

After documentation of 1722 patients, The GPG 1 was completed at the end of 1993. According to the study protocol, 426 patients were followed up until 1995 (> 80%).

Key results included, that after type C pelvic ring injuries, 80% anatomical healing was observed, but after two years only 60% of these patients had a good clinical outcome [9, 11, 12]. This gray area remained to be analyzed. Another result was that the previously trivialized type A injuries led to long-term complaints in 50% [9, 11, 12]. This was inexplicable at that time, but gives already a first indication of the still unknown geriatric insufficiency fractures at that time.

Based on the chosen study design several specific questions could not be answered adequately, e.g. the mentioned discrepancy between clinical and radiological results in type C pelvic ring injuries, the influence of concomitant nerve damage on long-term results, causes of poor results in acetabular fractures etc.

**Fig. 4:**
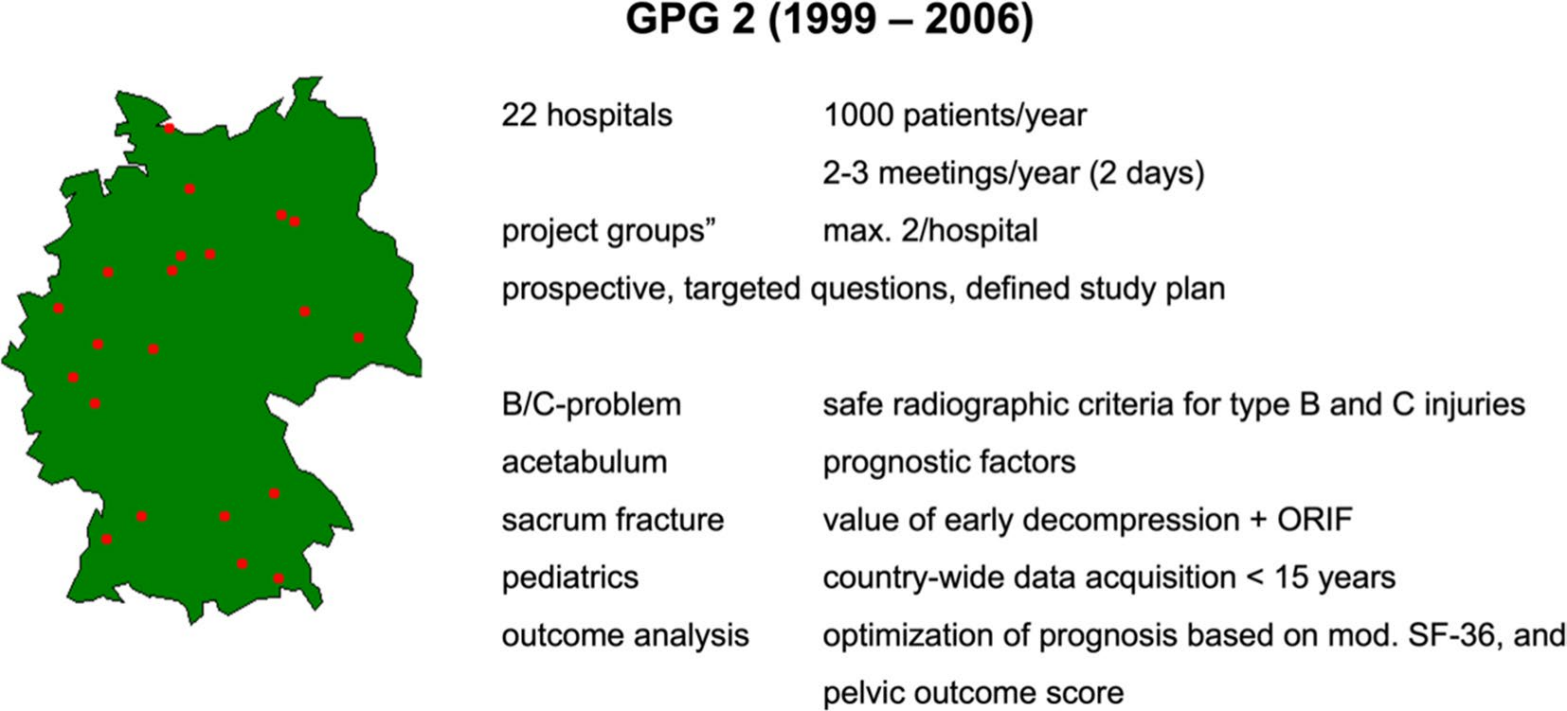
Target questions and topics of the GPG 2

The data analysis was funded by AO-International and published in a bilingual book (Fig. 4).

### The 2nd German Pelvic Group (GPG 2) (chairman: Prof. T. Pohlemann)

During the data evaluation phase of the GPG 1, regular meetings took place, but no further data were collected.

In 1997, the steering committee of the DGU approved the GPG 2. With now 22 hospitals, a register was set up and continuously maintained. The structure of the database was redesigned in a more modular way, as not all data were required for every patient. A basic data set was supplemented by temporary study modules (sacrum fractures, complex trauma, pediatric trauma and pelvic ring stability assessment).

As the machine reading of the data collection forms could never be achieved due to financial reasons, the data in the individual hospitals were recorded in database clones and transferred to the central database on simple diskettes (floppy disc).

On the initiative of Professor Heinrich Reimann, Head of Braunschweig Trauma Hospital) a funding of 50.000 DM was initiated for a digital image exchange network and implementation for medical video conference discussions. The experiences gained here had significant influence into the later designed Tele-Cooperation of the DGU.

The GPG 2 was founded in 1997 and accredited between 1999 and 2006.

**Fig. 5:**
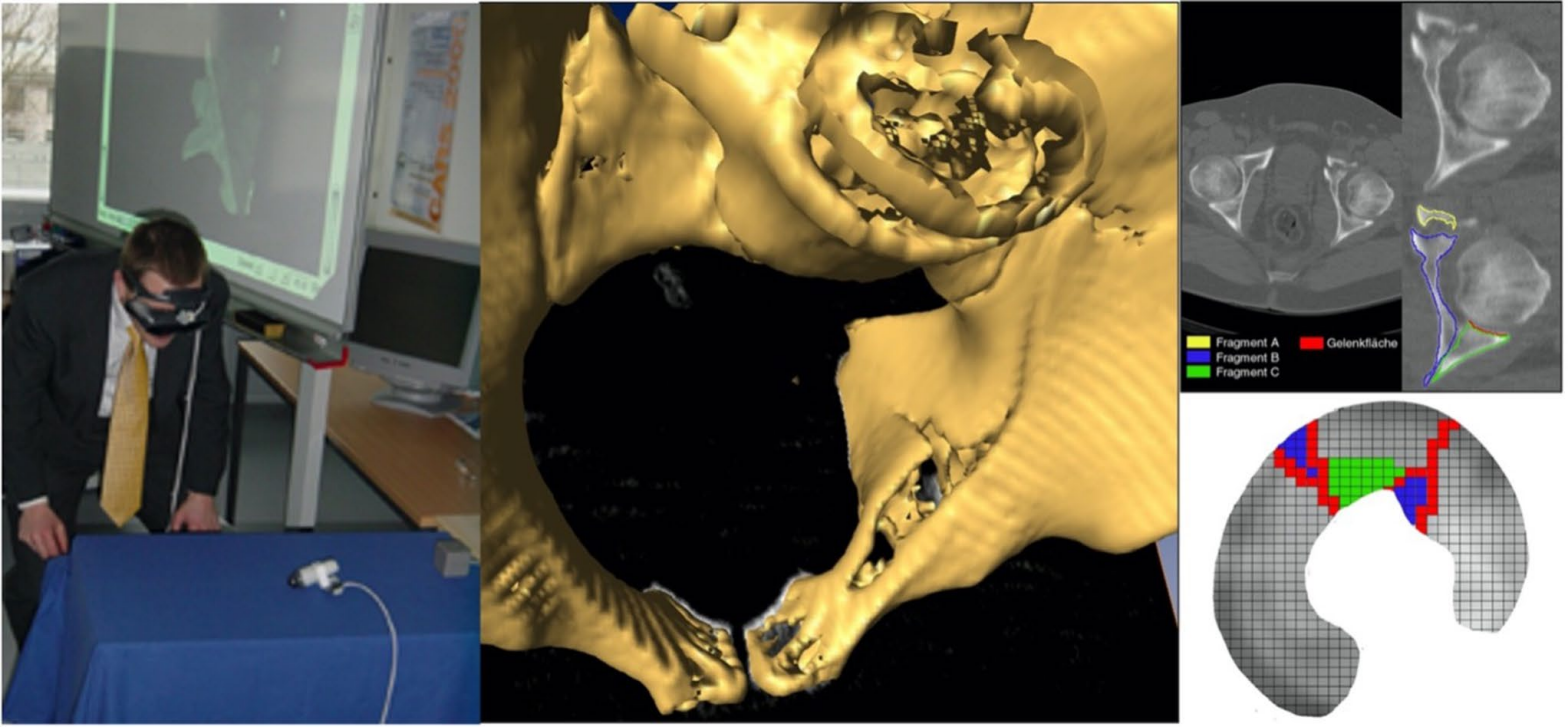
Special focus on new options of acetabular fracture visualization

The aim of the GPG 2 was to answer specific questions (Fig. 4) based on the experience of the GPG 1 in a prospective analysis using a defined study plan:

- B/C problem: development of reliable radiological classification criteria leading to better treatment indications; radiological analysis of stability transition from type B to type C injuries; analysis of factors that cause C instability; radiological markers for secondary dislocation.
- acetabulum: analysis of prognostic factors (e.g. fracture type, additional injuries, degree of dislocation, quality of reduction, comminution zones, marginal impactions, intra-articular fragments) on the long-term outcome after acetabular fractures (osteoarthritis rate, femoral head necrosis rate, THR rate) on the long-term results.
- sacral fractures: analysis of the value of early sacral spinal decompression with subsequent stabilization, prognosis assessment.
- pediatric fractures (< 15 years): comprehensive descriptive analysis of all pelvic ring and acetabular fractures in children.
- outcome: extended analysis of the long-term outcome of unstable pelvic injuries and acetabular fractures using the German version of the SF36 and the GPG 1 Outcome Score, enabling a more precise prognoses for individual pelvic and acetabular fractures.
- evaluation of new visualization techniques for acetabular fractures in collaboration with the Technical University Braunschweig (Fig. 5).

### The 3rd German Pelvic Group (GPG 3; chairman: Prof. U. Culemann)

The concept of data transfer via diskette became impractical and parallel the Internet possibilities were stabilized. As the first database of the DGU, the GPG established an Internet-based database in collaboration with the Maurice E. Müller Documentation Institute in Berne, Switzerland. The international experience and the security concept were convincing and with the aim of international expansion, the Swiss location with university connections seemed to be the best address. The transformation of the data structure was complex. However, a synthesis was found between master data and special studies. Accepted redundancies later led to considerable difficulties. In addition to the register research, the GPG continued to deal intensively with topics of further training in the development of innovative surgical techniques such as navigation-assisted surgery.

From 2002, an additional forum was created with the Homburg/Saar Pelvic Courses that allowed intensive discussion of current topics on pelvic and acetabulum surgery. By the anniversary year of 2022, over 1300 medical colleagues and around 100 nursing staff had been trained in the topic of pelvic and acetabular fractures.

Various scientific publications were published based on the pelvic register. For example, an improvement of the mortality rate after pelvic fractures was observed, the demographic shift and the peculiarities of geriatric fractures were identified, and there was also a clear change in the indication, choice of approach and surgical strategy for acetabular fractures. Additionally, the mortality rate of complex pelvic injuries was reduced with standardized acute measures, but still remained at a level of around 20%. Long-term analyses of acetabular fractures in average 11 years after trauma revealed, that 80% of the joints were still well functioning [1, 7, 13, 14, 16].

### Recent developments

Currently, data entry is carried out by the Academy of Trauma Surgery (AUC). The architecture of the database has been aligned with the DGU trauma register. A general input mask leads to the universal recording of all trauma data. If specific criteria are met (participation of the clinic, inclusion criteria, etc.), additional recording in the pelvic register is possible. With this future-proofed structure, the DGU has taken a further step to carry out its own scientifically based analyses of trauma treatment in Germany and thus will be able to react in a controlled manner to treatment reality. This simplification further increases the number of participants in the GPG. The new European version on data protection regulation from 2019 led to further uncertainty about deficits in data quality. However, the increasing professionalization in local data management also offered possible solutions, which increased the rate of patient consent to the use of data again. After 32 years, the GPG is still active without any noticeable signs of fatigue. The GPG is working on current future topics such as automated image recognition for classification using artificial intelligence methods, minimally invasive video-assisted procedures, navigation devices and new approaches to data analysis. The GPG is currently concentrating on a comprehensive S2 guideline and is thus supporting the diverse efforts to improve the quality and safety of care.

Recent aims of the GPG include:

- expansion of the database to the international register as well as national expansion and acquisition of additional hospitals.
- expansion of the register to the world’s largest register to answer specific and detailed questions about pelvic injury care.
- integration of non-maximum care providers into the register in order to reflect the entire reality of care for pelvic ring and acetabular fractures.

Since the founding of the GPG 30 years ago, a total of around 20,000 data sets on pelvic ring and acetabulum fractures have been recorded.

Between 2004 and 2002, approximately 19.800 cases were integrated into the Pelvic registry and approximately 45 scientific papers were published. The GPG consists of 117 members and 31 hospitals which are authorized to use the register. 11 additional hospitals are in the process of contractually registering to participate in the register. The aim is to further increase the number of participating hospitals, also with the aim of acquiring more regional and national hospitals in order to provide a better overall view of the treatment of pelvic ring and acetabulum fractures.

In the DGU Board meeting in June 2024 the Section Pelvic and Acetabulum was approved and upgraded in a standing section of the German Society of Trauma Surgery (DGU).

## Conclusion

In 1990, the GPG was initiated by the German section of the AO-International and the DGU. Under the initial leadership of Professor Harald Tscherne and Tim Pohlemann a prospective study with data analysis of a total of 1722 patients over three years at ten participating hospitals was initiated and formed the basis for a detailed analysis of the national treatment standard in pelvic and acetabular surgery. This led to the development of a nationwide pelvic registry, which is chaired presently by the Section Pelvic and Acetabulum, operated by the German Academy of Trauma Surgery (AUC). Based on this initiative, various clinical research projects were initiated and national networks were formed for professional exchange.

The database is currently a part of the National DGU Trauma registry and adds important information to a polytrauma situation, but also can act as stand-alone for specific P/A questions. An additional focus has always been placed on post-graduate training during all meetings, including hands-on offerings. By this the Group acted as a motor for many surgical and structural improvement, e.g. after detection of the impact of demographic changes to the nature of resulting osteoporosis-based injuries. Also, specific multicenter studies could proof the quality of care after P/A injuries in Germany, the value of close networking within the Trauma Networks DGU to find optimal treatment options in specialized centers. Of interest is certainly a study about the long-term outcome after acetabular surgery in a multi-center, multiple surgeons setting, which proofed comparable long-term results to the classic single surgeon series by Letournel and Matta [3–6, 15]. Thus, the GPG is an important pillar in the German Trauma Societies commitment of improving the standard of care after injuries to the pelvis and acetabulum, from initial treatment to rehabilitation.

## Data Availability

No datasets were generated or analysed during the current study.
